# Book Review of Clinical MR Physics: State‐of‐the‐Art Practice By Ho‐Ling Anthony Liu and Xiaohong Joe Zhou (Medical Physics Publishing, Inc. pp. 1‐561. June 2025. ISBN: 9781951134365)

**DOI:** 10.1002/acm2.70520

**Published:** 2026-02-27

**Authors:** Donglai Huo

**Affiliations:** ^1^ Department of Radiology University of Colorado Anschutz Medical Campus Aurora Colorado USA

## 1 OVERVIEW

As stated in the preface, *Clinical MR Physics: State‐of‐the‐Art Practice* is an AAPM (American Association of Physicists in Medicine) monogram, curated from the successful 2025 AAPM summer school hosted at University of Denver from June 19–24, 2025. The summer school (and the book) are both designed for clinical MR professionals, including both diagnostic and therapy physicists, MR scientists, MR engineers, and trainees who want to learn more about clinical MR applications.

## 2 ORGANIZATION

The book, following the structure of the summer school and totaling 561 pages, consists of 6 parts: “Essentials of Clinical MR Physics”, “Facility Planning, Performance Testing, and Accreditation”, “MR Safety”, “MR Protocols”, “Quantitative and Advanced MR”, and “Emerging Technologies”. The book's 29 chapters were authored by 36 MR field experts, including contributions from the two editors and five associate editors, who also provided overall editorial leadership.

## 3 CHAPTER CONTENTS

Part 1 of the book starts with the very basic concept of MR, from NMR (Nuclear Magnetic Resonance) to MRI (Magnetic Resonance Imaging) in Chapter 1, and naturally leads to the MR pulse sequences (Chapter 2), where gradient echo, spin echo, inversion recovery, echo planner imaging, and MR angiography were introduced. In Chapter 3, the authors discuss acceleration techniques, such as parallel imaging, and advanced applications such as diffusion weighted imaging. Some of the topics are further illustrated in later chapters. The book focuses on clinical applications; therefore, MR image quality is covered in Chapter 4, and in Chapter 5, it provides a nice summary of MR artifacts.

Moving to Part 2, this section is designed solely for clinical MR physicists. The authors share experiences and design principles of MR system and site planning (Chapter 6), MR performance testing (Chapter 7) and discuss MR physicist role in Accreditation (Chapter 8).

Part 3 continues with clinical MR issues, focusing on the importance of MR safety. After a basic MR safety introduction in Chapter 9, separate safety topics are discussed in detail for B0 (Static Field) in Chapter 10, Gradient in Chapter 11, and B1 (Radio Frequency) in Chapter 12. The clinical challenging problem of scanning patients with implants is well explained in 3 Chapters: where Chapter 13 focuses on RF interaction, Chapter 14 focuses on the MR conditional labeling, and Chapter 15 gives an overview of related standards and regulations. Finally, Chapter 16 concludes Part 3 with the discussion of implementing MR safety programs.

Part 4 of the book focuses on clinical MR protocols. Neuro, Breast, Body, and Musculoskeletal MRI protocols are discussed individually in Chapters 17–20. While it is impossible to cover every MR protocol–related topic in one chapter, the authors did a great job balancing between basic principles, clinical considerations, and real protocol optimization examples.

Going into Part 5, advanced MR applications are discussed here. These applications are technically mature, actively researched, and increasingly being incorporated into routine clinical exams. In Chapter 21, MR relaxometry is reviewed systematically, covering T1 mapping, T2 mapping, MR fingerprinting, and synthetic MRI. Fat quantification and iron quantification techniques are discussed in Chapter 22. Diffusion and perfusion imaging, from principle to applications, are covered in detail in Chapters 23 and 24. Chapters 25 and 26 provide a detailed review of fMRI and MR spectroscopy.

The last section of the book, Part 6, focuses on the new MR techniques. Chapter 27 discusses the hardware inventions, including non‐traditional B0‐field MR systems (high‐field, low‐field, and portable) and high‐performance gradient systems. A fast‐developing field, MR in radiation therapy, is covered in Chapter 26. While traditionally MR physicists work more within radiology, MR becomes increasingly prevalent in radiation oncology, and the unique opportunities and challenges are well‐explained in this chapter. Finally, in the last chapter, Chapter 29, AI technology is covered, and its impacts on MRI reconstruction, image processing and image synthesis are discussed.

## 4 SUMMARY

MRI is no doubt one of the most complicated imaging modalities in diagnostic imaging. Limited by the scope, although having a very solid review, this book is not designed for beginners to learn MR. Other books, such as *MRI: Basic Principles and Applications (5th ed., Wiley, 2015) by Dale, Brown, and Semelka* could serve as a better textbook. Similarly, for MR engineers and researchers who are looking for a deeper dive on MR sequences and reconstructions, books such as *The Handbook of MRI Pulse Sequences (Elsevier, 2004) by Bernstein, King, and Zhou* and *Principles of Magnetic Resonance Imaging: A Signal Processing Perspective (Wiley‐IEEE Press, 1999) by Liang and Lauterbur* could be more helpful. However, for clinical MR physicists, this book provides an extensive, up‐to‐date reference addressing all key clinical MRI topics.

Although all diagnostic physicists received basic MR safety and MR equipment testing training, not all physicists are comfortable with MRI as much as other X‐ray‐based modalities. This book offers an excellent opportunity to bridge MRI theory with real‐world clinical challenges. In particular, for MR safety and regulatory requirements, this book offers the most up‐to‐date and thorough coverage. While primarily intended for clinical MR physicists, this book could also serve as an excellent reference for other physicists involved in clinical MR applications. For physicists pursuing MRI board certifications—such as those offered by the American Board of Medical Physics or the American Board of Magnetic Resonance Safety—this book is an excellent resource, written by experienced MR physicists with first‐hand expertise.

